# Two-Photon Microscopy for Non-Invasive, Quantitative Monitoring of Stem Cell Differentiation

**DOI:** 10.1371/journal.pone.0010075

**Published:** 2010-04-16

**Authors:** William L. Rice, David L. Kaplan, Irene Georgakoudi

**Affiliations:** Department of Biomedical Engineering, Tufts University, Medford, Massachusetts, United States of America; CNRS, France

## Abstract

**Background:**

The engineering of functional tissues is a complex multi-stage process, the success of which depends on the careful control of culture conditions and ultimately tissue maturation. To enable the efficient optimization of tissue development protocols, techniques suitable for monitoring the effects of added stimuli and induced tissue changes are needed.

**Methodology/Principal Findings:**

Here, we present the quantitative use of two-photon excited fluorescence (TPEF) and second harmonic generation (SHG) as a noninvasive means to monitor the differentiation of human mesenchymal stem cells (hMSCs) using entirely endogenous sources of contrast. We demonstrate that the individual fluorescence contribution from the intrinsic cellular fluorophores NAD(P)H, flavoproteins and lipofuscin can be extracted from TPEF images and monitored dynamically from the same cell population over time. Using the redox ratio, calculated from the contributions of NAD(P)H and flavoproteins, we identify distinct patterns in the evolution of the metabolic activity of hMSCs maintained in either propagation, osteogenic or adipogenic differentiation media. The differentiation of these cells is mirrored by changes in cell morphology apparent in high resolution TPEF images and by the detection of collagen production via SHG imaging. Finally, we find dramatic increases in lipofuscin levels in hMSCs maintained at 20% oxygen vs. those in 5% oxygen, establishing the use of this chromophore as a potential biomarker for oxidative stress.

**Conclusions/Significance:**

In this study we demonstrate that it is possible to monitor the metabolic activity, morphology, ECM production and oxidative stress of hMSCs in a non-invasive manner. This is accomplished using generally available multiphoton microscopy equipment and simple data analysis techniques, such that the method can widely adopted by laboratories with a diversity of comparable equipment. This method therefore represents a powerful tool, which enables researchers to monitor engineered tissues and optimize culture conditions in a near real time manner.

## Introduction

The goal of tissue engineering is the development of functional tissue equivalents for the repair or replacement of that lost to damage or disease [1]. Tissues are constructed via a complex process in which non-terminally differentiated cells, such as human mesenchymal stem cells (hMSCs), are combined with a biomaterial scaffold and induced to differentiate into a functional tissue via a variety of physical and/or chemical stimuli. Many factors influence hMSC proliferation and differentiation, including soluble factors added to the culture medium [2], [3], oxygen tension[4], culture temperature [5], scaffold design[6], mechanical and electrical properties [7]. In addition to the culture environment, the choice of stimuli as well as the timing and order of their application is critical for the successful development of the desired tissue [8]. Therefore, design and optimization of culture protocols requires close monitoring of multiple tissue characteristics during the development of the engineered tissue. Specifically of interest are tissue viability, cell proliferation, metabolic state, differentiation, morphology and production of extracellular matrix molecules and changes in the original biomaterial matrix.

These characteristics are currently monitored using methods such as electron microscopy, histochemistry or colorimetric metabolic assays that, because of their need for processing, are destructive in nature and, therefore, incompatible with the continued experimental use of the sample. Recently, researchers have begun to assess new non – destructive technologies that permit repeated measurements of the same sample as tissue development progresses. Optical techniques such as confocal and multiphoton microscopy (MPM) are emerging as powerful methods for non-invasive characterization and monitoring of engineered tissues [9], [10].

Multiphoton microscopy has several advantages over confocal and traditional fluorescence microscopy, including decreased out of focus photodamage, increased imaging depths and intrinsic optical sectioning [11]. The use of near infrared excitation wavelengths allows for the imaging of tissues using endogenous contrast through two photon excitation of fluorescence (TPEF) and second harmonic generation (SHG) without the need for ultraviolet light which can be deleterious to tissues [12], [13]. The greatest contributors of cellular TPEF are predominantly mitochondrially localized NADH and flavoproteins (FP) [13], while extracellular fibrous collagens result in SHG [14].

The use of TPEF has enabled researchers to characterize cell viability, morphology, and proliferation in both excised and engineered tissues [15]–[18]. SHG imaging has been used to monitor cellular collagen deposition [19], extracellular matrix remodeling during culture [20] as well as the effects of tissue preservation techniques[16].

In this study, we applied TPEF and SHG imaging to monitor the progress of hMSC cultures stimulated with adipogenic and osteogenic differentiation factors and maintained under atmospheric or hypoxic (5%) oxygen concentrations. Based on multi-spectral analysis approaches, we demonstrate that the major endogenous chromophores that contribute to the observed TPEF signal from hMSCs include NAD(P)H, flavoroteins and lipofuscin. We present a method to quantify the contributions from each of these fluorophores and assess dynamically over time changes in lipofuscin accumulation and metabolic activity as stem cells differentiate. In addition, we monitor the production of fibrous collagens using SHG. We identify distinct patterns of changes in these quantitative biomarkers as a function of differentiation pathway (i.e. adipogenic vs. osteogenic) and of ambient oxygen concentration.

## Results

### Identification of intrinsic cellular fluorophores

**Figure 1:**
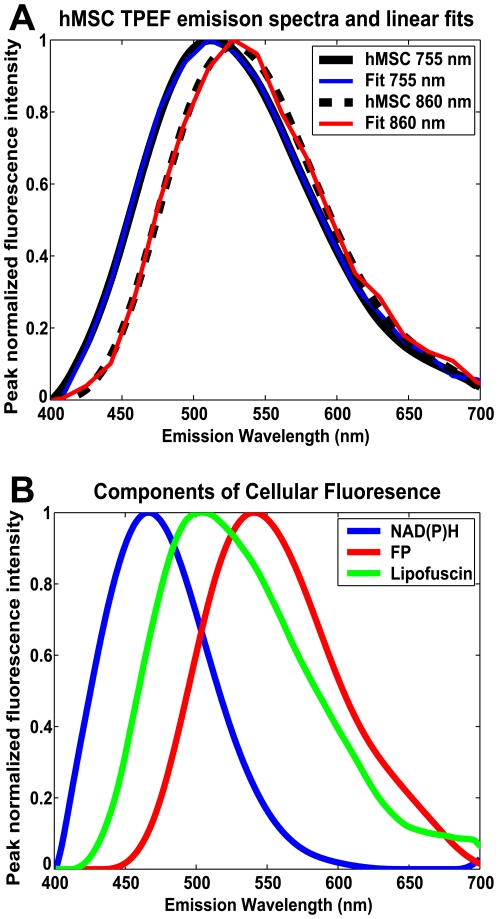
Components of hMSC TPEF emission spectra. (A) hMSC TPEF emission spectra collected at either 755 nm or 860 nm excitation with corresponding fits by the ALS script. (B) linear unmixing methods reveal the contributing cellular fluorophores to be NAD(P)H, Lipofuscin and FP. When components of intrinsic cellular fluorescence are used to produce the fits displayed in panel A the NAD(P)H component does not contribute to cellular spectra excited at 860 nm.

**Figure 2:**
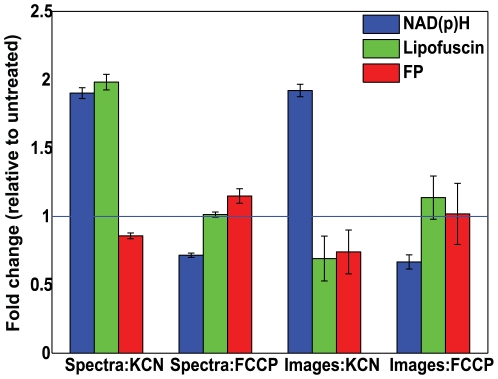
TPEF analysis of hMSCs following KCN or FCCP treatment. The inverse fluctuations of NAD(P)H and FP fluorescence in response to KCN or FCCP treatment were detectable via either TPEF emission spectra excited at 755 nm, or TPEF images excited at 755 nm and 860 nm. For each method, means are displayed relative to values before treatment with standard error bars. Spectral data from multiple microscopic fields (N = 9) were used for the analysis of the TPEF emission spectra while the results from TPEF images were calculated on a cell by cell basis from multiple image sets (N = 42).

**Figure 3:**
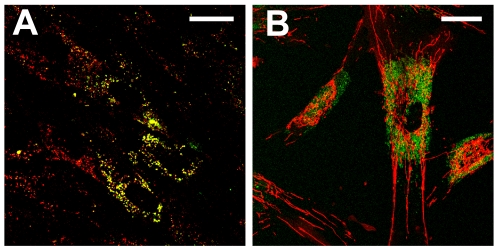
Lysosomal localization of lipofuscin. Lipofuscin autofluorescence (green channel) is co-localized with (A) Lysotracker red (red channel) and not (B) Mitotracker Orange (red channel). Co-localization is indicated by the yellow color in panel A. Note that not all lysosomes contain lipofuscin. Bar  = 30 µm.

To identify the intrinsic cellular fluorophores excited in the hMSCs at 755 nm and 860 nm, TPEF emission spectra were collected from 400 nm to 700 nm for each excitation wavelength (Figure 1A). Linear unmixing of the emission spectra revealed three components, shown in Figure 1B, that are consistent with the literature values for NAD(P)H, lipofuscin and flavins [13], [21], [22]. The flavin (FP) and lipofuscin component spectra were present at both 755 nm and 860 nm excitation, while the NAD(P)H component spectrum was only present with 755 nm excitation and was blue shifted compared to an aqueous NADH solution [13]. In Figure 1A, representative cellular emission spectra are shown along with fits to the spectra using the components identified from the spectral decomposition. The combined unmodelled variance for these two fits as reported by the fitting algorithm was 0.04%. To confirm the identity of the NAD(P)H and FP components and our ability to detect changes in their levels of fluorescence, hMSCs were treated with either 4 mM KCN or 4 µM FCCP. The results of KCN and FCCP treatment detected by either linear unmixing of TPEF emission spectra excited at 755 nm (N = 9 fields) or the combined analysis of TPEF images excited at both 755 and 860 nm (N = 42 cells) are shown in Figure 2. As expected, addition of KCN to the culture medium results in a relative increase in NAD(P)H fluorescence and a decrease in FP levels while FCCP treatment shows a decrease in NAD(P)H and a slight increase in FP fluorescence. Further confirmation of the identity of the lipofuscin component was obtained by its lysosomal localization as determined by the co-localization of the bright autofluorescence and lysotracker, but not mitotracker (Figure 3).

### Monitoring of hMSC differentiation with TPEF imaging

**Figure 4:**
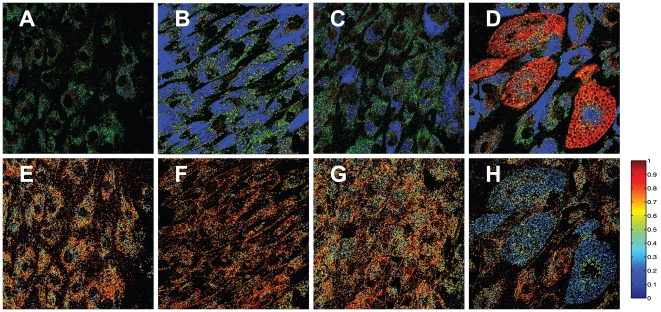
Concentration and redox ratio images of hMSCs. Representative false color concentration images (A–D) and corresponding redox ratio images (E–H) from HP (A,E), NP (B,F), NO (C,G) and NA (D,H). In the concentration images, NAD(P)H, FP and lipofuscin concentrations are represented in the red, green and blue channels, respectively. The redox ratio, calculated as [FP]/([FP]+[NAD(P)H]) is inversely proportional to metabolic activity with blue colors representing high levels of metabolic activity and orange colors representing low levels of metabolic activity. Bar  = 50 µm.

Analysis of TPEF emission spectra allows for the comparison of relative levels of cellular fluorophores from one microscope field to another, while the increased image quality of the TPEF images enables these measurements to be made on a cell by cell basis. The acquisition of TPEF images is also more efficient, requiring fewer scans of the microscope field than when TPEF emission spectra are collected (4 vs 20), delivering less average laser power in the process and thus reducing the risk of damage to the sample, Nevertheless, in this study no changes in cell morphology or other adverse effects were observed from the cells with either modality. As described in the materials and methods, TPEF images excited at 755 nm and 860 nm are acquired from the same hMSC cultures over 16–21 days and analyzed for changes in NAD(P)H, lipofuscin and FP levels. As shown in Figure 4, false color images can be created to illustrate changes in which NAD(P)H, FP and lipofuscin relative concentrations are depicted by the red, green and blue channels, respectively. Alternatively, these images, which retain information detailing cell density, orientation and morphology, can be displayed as redox ratio-based images, in which cellular metabolic activity is represented on a red to blue color scale. In Figure 4(E–H), redox ratio images indicate differences in metabolic activity between cells in differentiation medium and those in propagation medium, with blue representing high activity and orange representing low activity. The heterogeneity of cell responses to differentiation stimuli within the same treatment group is well represented by panels D and H of Figure 4. In these panels, cells undergoing adipogenic differentiation can be clearly identified by an enlarged area with multiple voids in the fluorescence signal due to lipid droplet accumulation. The increased contribution of NAD(P)H to the cellular fluorescence is indicated by the red coloring of these differentiating cells in Figure 4D. In Figure 4H, increased metabolic activity highlights the differentiating cells with a blue redox ratio, while others in the same dish retain a less active, more orange-red redox ratio. Such striking intra- sample differences are not observed in other treatment groups.

### NAD(P)H, FP, and Redox Ratio

**Figure 5:**
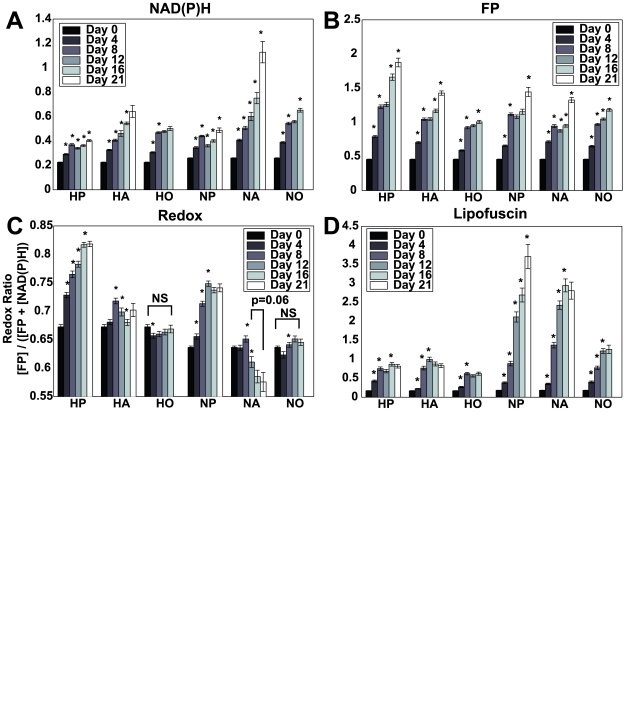
Quantitative monitoring of hMSC intrinsic fluorescence. The mean concentration (in relative fluorescein concentration units) of (A) NAD(P)H, (B) FP, and (D) Lipofuscin per cell was monitored over 16 or 21 days and is presented with standard error. To evaluate changes in metabolic activity, a redox ratio was calculated as [FP]/([FP]+[NAD(P)H) and is shown in panel C as the mean, per cell redox ratio with standard error. Statistical difference in mean values compared to the previous time point are indicated by an asterisk. NS indicates a lack of statistical significance while the mean redox ratios on days 12 and 21 for the NA group are only different with a p value of 0.06. N≥44 cells per time point.

Mean NAD(P)H and FP levels are determined on a cell by cell basis (N≥44) from TPEF images acquired at 755 nm and 860 nm over 16 or 21 days of culture. In Figure 5A and 5B, the mean NAD(P)H and FP concentration per cell is presented from hMSC cultures in either propagation, osteogenic or adipogenic medium maintained under hypoxic (5% oxygen) (HP, HO, HA) or normoxic (20% oxygen) (NP, NO, NA) conditions. In each treatment group, significant increases in the concentrations of NAD(P)H and FP are observed in comparison to the first day of imaging throughout the experimental period.

Stem cells cultured in hypoxic conditions exhibit relatively lower concentrations of NAD(P)H than those cultured in normoxic conditions at every time point. hMSCs in propagation and osteogenic differentiation medium experience an increase in NAD(P)H concentration on days 4 and 8 regardless of oxygen concentration. This initial increase is followed by either a decrease in measured NAD(P)H concentration (propagation medium) or no change (osteogenic medium) on day 12. For cells in propagation medium, the NAD(P)H concentration does not surpass that measured on day 8 until day 21, while in the osteogenic differentiation groups the measured NAD(P)H is greater on day 16 in comparison to day 8. hMSCs in adipogenic differentiation medium (HA, NA) maintain increases in NAD(P)H levels at each time point over the entire culture period.

Similarly, FP concentration increases are observed in each treatment group during the experiment (Figure 5B), with cells in propagation medium at 20% oxygen (HP) exhibiting the greatest amount at each time point. For hMSCs in osteogenic differentiation medium, the mean FP concentration is significantly lower at 5% oxygen than 20% oxygen for each time point. Conversely, the hMSCs in adipogenic medium under hypoxic conditions have significantly more FP than their normoxic counterpart after day 4.

In order to compare the metabolic activity of hMSCs under different culture conditions, the mean redox ratio per cell was computed for each time point. As shown in Figure 5C (and Figure 4E–H) distinct patterns of redox ratio changes are observed that depend not only on the differentiation status of the cells, but also on the specific differentiation lineage. Specifically, hMSCs in propagation medium develop a higher redox ratio than those in differentiation medium, with the highest redox ratio achieved on day 16 (5% oxygen) or on day 12 (20% oxygen). Cells in osteogenic differentiation medium at 5% and 20% oxygen exhibit an initial decrease in the redox ratio on day 4, followed by a consistent increase, with the mean values achieving statistically similar values to those of day 0, by day 8 (in the case of normoxia) or day 12 (in the case of hypoxia). Interestingly, hMSCs in adipogenic differentiation medium exhibit an inverse trend, consisting of an initial increase in redox ratio followed by a decrease. Cells cultured in this medium at atmospheric oxygen experience the greatest decrease in redox ratio of all the treatment groups over the length of the experiment. Following a significant increase (compared to days 0 and 4) in redox ratio on day 8, NA cells exhibit a redox ratio that is significantly lower than day 0 for the rest of the experiment. In comparison, when hMSCs in adipogenic medium are maintained in 5% oxygen, they experience a significant increase in redox ratio on day 8 (compared to days 0 and 4) followed by subsequent decreases on days 12–21; however, the level of decrease is not as pronounced as for the NA group, with values on day 16 that are not different than those for day 0.

### Lipofuscin

Figure 5D shows the evolution of lipofuscin concentration for each treatment group throughout the experiment. On culture day 0 there is no significant difference in the mean per cell lipofuscin concentration regardless of oxygen tension. By day 4, NP, NA and NO cells have significantly higher lipofuscin levels than HA and HO. This trend continues as hMSCs maintained in 20% oxygen experience a greater accumulation of lipofuscin than those in hypoxic conditions. Day 4 represents the only time point at which hMSCs from the hypoxia propagation group (HP) have greater lipofuscin levels than those maintained in normoxia. Over all treatment groups, the least amount of lipofuscin is observed in hMSCs in osteogenic differentiation medium under hypoxic conditions.

### SHG imaging of collagen

**Figure 6:**
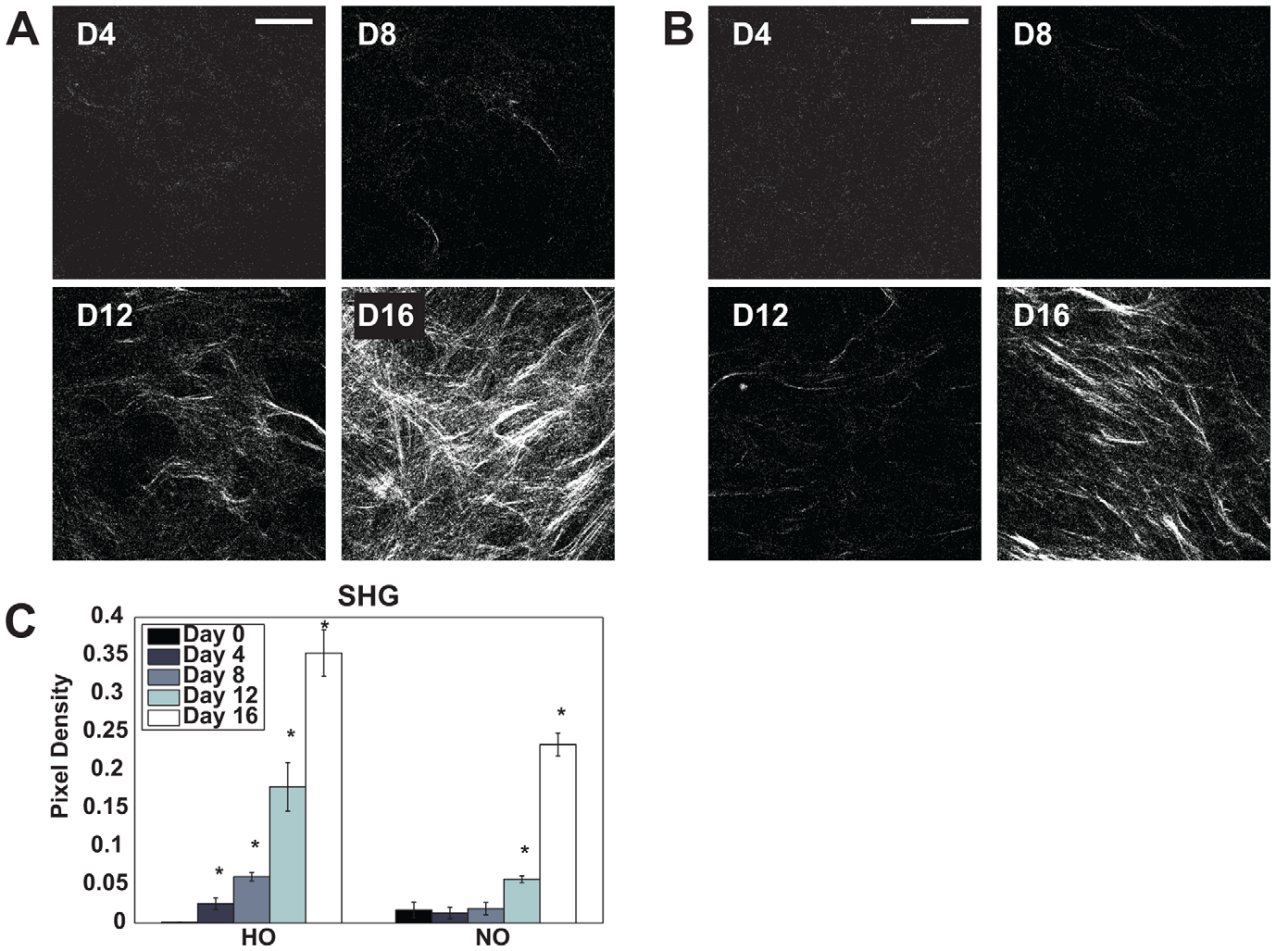
SHG evaluation of fibrous collagen. The gradual deposition of fibrous collagens is apparent in SHG images acquired on days 4, 8,12 and 16 from hMSCs in osteogenic medium at (A) 5% oxygen and (B) 20% oxygen. Quantitative evaluation of pixel density in SHG images reveals that collagen deposition evolves earlier for HO conditions than for NO cells. N = 4. (Bar = 50 µm).

SHG images, as shown in Figure 6, reveal the deposition of fibrous collagens by cells undergoing osteogenic differentiation. Analysis of SHG images indicates accumulation of type I collagen in the HO group significantly earlier than in the NO group (Figure 6, N = 6). Specifically, SHG pixel density from HO cells was significantly increased at each time point (compared to the previous day), while no significant increase was observed from NO until day 12. No collagen deposition was observed in the propagation and adipogenic differentiation groups.

### Alizarin red mineralization assay

**Figure 7:**
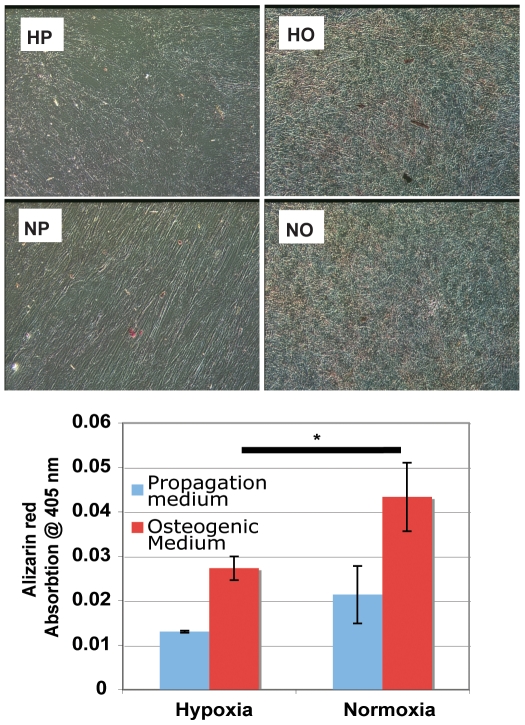
Alizarin Red Mineralization Assay. Only small, diffusely distributed mineral deposits are detected in phase contrast images of stained cultures that are more prominent in hMSCs in osteogenic differentiation than in propagation media. Quantitative analysis by absorption at 405 nm of eluted alizarin red stain reveals significantly more staining of NO cells than HO (denoted by asterisk). N = 6.

To assess the level of mineral deposition, hMSCs maintained in osteogenic differentiation medium were stained with an Alizarin red solution on day 16. Qualitatively, the staining appeared darker for both HO and NO compared to HP and NP. However, no obvious mineral nodules were observed under phase contrast microscopy (Figure 7). Quantitative analysis of the eluted Alizarin red stain revealed significantly more staining in NO compared to HO hMSCs (Figure 7, N = 6).

### Oil Red - O for lipid accumulation

**Figure 8:**
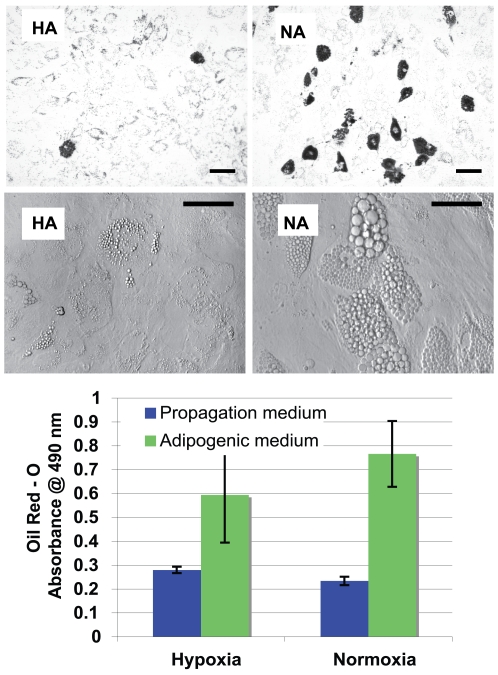
Oil Red - O Staining for lipid droplets. hMSCs maintained in adipogenic differentiation medium were stained with Oil Red - O to highlight the lipid contents of cells (top images, bar  = 100 µm). Higher magnification transmission images of HA and NA (bottom images, bar = 50 µm) before staining reveal larger, more numerous lipid droplets in hMSCs cultured in adipogenic differentiation medium at 20% oxygen. Quantification of eluted Oil Red - O by absorption of 490 nm reveals an increase in total Oil Red - O staining amongst HA or NA cells, but the differences are not statistically significant. N = 6.

Comparison of the bright field images in Figure 8 of HA and NA hMSCs reveal differences in lipid droplet accumulation with larger, more numerous lipid droplets visible on the final day of culture from those at 20% oxygen concentration. Oil Red - O staining for lipids allows for the visualization of a greater frequency of large lipid droplet containing cells in the NA group while most of the cells in the HA group appear to contain small droplets that are stained with the solution. Subsequent elution of the stain and quantitative analysis via absorption reveals that observed differences in the amount of Oil Red - O staining between HA and NA groups do not reach significance (N = 6).

### RT-PCR

**Figure 9:**
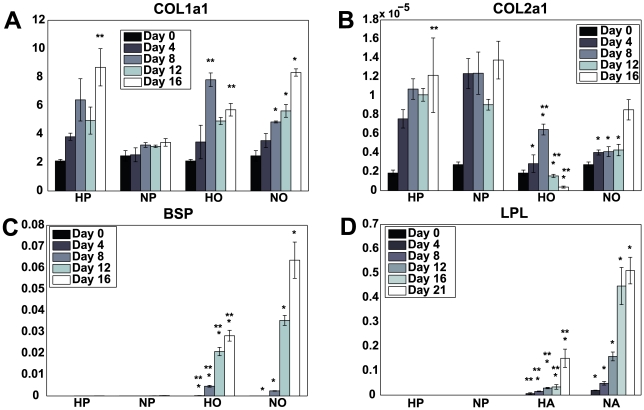
RT-PCR. Transcript levels of COL 1a1, COL 2a1, BSP and LPL were assessed for each imaging time point relative to GAPDH. Statistically significant differences in mRNA transcript levels are denoted by a single asterisk (in comparison to the propagation medium at the same oxygen tension) or by a double asterisk (significance in comparison to cells of the same medium condition, at 20% oxygen tension). N = 4.

As a confirmation of the effects of the differentiation medium, mRNA transcription levels of collagen 1a1 (COL 1a1), collagen 2a1 (COL 2a1), bone sialoprotein (BSP) and lipoprotein lipase (LPL) were determined relative to glyceraldehyde 3-phosphate dehydrogenase (GAPDH) for each imaging time point (N = 4). As shown in Figure 9A, COL 1a1 expression in HP cells was significantly higher on days 4–16 than day 0. While the changes in COL 1a1 expression from one time point to the next did not reach significance, the expression of COL 1a1 from HP cells on day 16 was significantly higher than on day 4.

For hMSCs in propagation medium at 20% oxygen, no significant changes in COL 1a1 expression were detected. For cells in osteogenic differentiation medium in hypoxic conditions, expression of COL 1a1 peaked at day 8 while the greatest expression observed from NO cells was on day 16. COL 1a1 expression on day 8 was significantly higher under hypoxic conditions than it was in normoxia while on day 16 expression in the normoxic group was significantly higher than that at 5% oxygen. COL 2a1 mRNA was detected at low levels in HO and NO groups with transcription levels significantly lower than cells in propagation medium. BSP mRNA expression after day 0 was greater in cells in osteogenic differentiation than those in propagation medium. Significant increases in BSP transcription were observed at each time point from HO cells until day 12, and from days 8 to 16 in NO cells. LPL transcription was significantly greater in NA cells in comparison to propagation medium (NP) on day 4, while a significant increase from HA cells (over that from HP cells) was not observed until day 8. For hMSCs in adipogenic differentiation medium, LPL transcript levels at 20% oxygen tension were significantly higher that those at 5% oxygen for all time points after day 4.

## Discussion

In this study, we demonstrate the utility of MPM imaging and spectroscopy as non invasive tools for monitoring hMSC cultures. Building on previous work from our laboratory, which established the use of TPEF for long term monitoring of stem cell biochemical status[17] we show that it is possible to isolate in TPEF images the individual fluorescence emission from the intrinsic cellular fluorophores NAD(P)H, FP and lipofuscin, by the careful selection of combinations of excitation wavelength and emission filters guided by the analysis of the TPEF emission spectra. TPEF emission spectroscopy allows for good spectral resolution, which is critical for determining the number and identity of the components of cellular TPEF [13]; however, low signal to noise ratio can limit overall image quality and acquisition of full spectra is time-consuming and can potentially result in greater levels of photodamage. TPEF images on the other hand are acquired through efficient band pass filters resulting in high image quality with high signal to noise ratio that can be collected with relatively few scans of the sample. As shown in Figure 4, these images retain detailed cellular morphology while providing biochemical and metabolic information. To our knowledge, this work represents the first application of MPM in which long term monitoring of hMSC differentiation reveals quantitative changes in cellular metabolic activity (via computed redox ratio) and oxidative stress (via lipofuscin) using entirely endogenous sources of contrast.

### Identification of the components of cellular TPEF

Most intracellular fluorescence originates from mitochondrial NADH and FP, while in some tissues it is possible to detect fluorescence from fluorophores such as vitamin A, lipofuscin and keratin with TPEF [9], [13], [23]–[25]. In our initial studies of hMSC intrinsic fluorescence, we observed bright, broad cellular autofluorescence [17].To identify the components of the hMSC intrinsic cellular fluorescence, we collected and analyzed TPEF emission spectra, establishing the spectral shapes of the contributing fluorophores. As shown in Figure 1B, linear unmixing of the TPEF emission spectra resulted in three component spectra that were consistent with NAD(P)H, lipofuscin and FP. The excitation and emission profile of the NAD(P)H component is similar to what has been reported in the literature: TPEF from NAD(P)H is detectable with 755 nm, but not 860 nm excitation [13] and a blue shift in the emission spectrum, when compared to aqueous NADH, reflects the dominant fluorescence contribution from protein bound NADH [24]. Our measurements lack the spectral resolution to differentiate between the bound and unbound forms of NADH or NADPH, therefore the NAD(P)H component represents all NADH and NADPH fluorescence [13]. Similarly, the FP component represents the majority of the cellular flavin fluorescence, which emanates from mitochondrial lipoamide dehydrogenase (LipDH) and electron transfer flavoprotein (ETF) [13].The shape of the FP emission spectrum is similar to that of aqueous FAD and can be excited at both 755 nm and 860 nm. The emission characteristics of lipofuscin as reported in the literature are variable between tissue types[21], [26] and can be similar in shape to that reported for vitamin A. However, the single photon absorption spectrum reported for vitamin A suggests weak two photon excitation at 860 nm [27], whereas the lipofuscin component can be excited at both 755 nm and 860 nm. The identity of the lipofuscin component was confirmed by its lysosomal localization (Figure 3A) [28].

Treatment of hMSCs with either KCN or FCCP further confirms the identity of these components and our ability to monitor the fluctuations of their fluorescence. Representing different sides of the cellular oxidation-reduction process, NAD(P)H is only fluorescent in its reduced form, while FPs are only fluorescent when oxidized. KCN, a powerful inhibitor of oxidative phosphorylation blocks the mitochondrial oxidation leading to an accumulation of reduced NAD(P)H and FP and thus an increase in detectable NAD(P)H fluorescence and a decrease in FP fluorescence. FCCP on the other hand destroys the mitochondrial proton gradient, and leads to an increase in NADH oxidation by ETF observable by a decrease in NAD(P)H fluorescence and a slight increase in FP fluorescence. As shown in Figure 2, the NAD(P)H and FP components respond as expected to KCN and FCCP treatment. The relatively muted response of the FP component especially after FCCP treatment is expected and has been reported by others [13], [29]. The observed fluctuations in relative (to pre treatment) lipofuscin concentration can be attributed to microscope field to field variations from cells containing little lipofuscin (day 0 Figure 5D) and not the effects of KCN or FCCP.

### Monitoring cellular activity with redox ratio

The metabolic activity of hMSCs is expected to fluctuate with proliferation, differentiation and senescence. In Figure 5A and 5B we have shown that it is possible to evaluate the relative changes in NAD(P)H and FP concentration from hMSCs under different culture conditions; however, it is difficult to draw conclusions about cellular metabolic activity from the analysis of the individual components, particularly because either component can be influenced by a number of biological processes. For instance Croce et al. have shown (as we do) increased NADH concentration in cells with higher metabolic rates [30] while others have shown that a decrease in NADH fluorescence is associated with increased metabolic activity[31]. Most cellular fluorescence emanates from mitochondrially localized NAD(P)H [32], [33] and FP, which are directly linked to cellular activity. Therefore, cellular metabolic activity estimated from the combined analysis of NAD(P)H and FP measurements is represented as a redox ratio[34]. The redox ratio, defined as [FP]/([FP] +[NAD(P)H]) has been used extensively in the literature and is presented as inversely proportional to cellular metabolic activity[29], [35], [36].

As shown in Figure 5C, statistically significant differences in cellular redox ratio develop between cells in proliferation medium and those undergoing differentiation. Undifferentiated hMSCs produce ATP primarily through glycolysis, while those undergoing differentiation switch to oxidative phosphorylation for their energy needs [37], [38]. We also observe a higher redox ratio in time point to time point comparisons from hMSCs in propagation medium under hypoxic conditions than we do from those maintained in 20% oxygen, which may be due to a greater reliance on glycolysis when in oxygen limited environments. Previous studies have used fluorescence based redox ratios to differentiate between cancerous tissues [36], [39] and found higher redox ratios correlated to high risk tumors which may also be due to a switch to a more glycolytic phenotype that has been termed the Warberg effect [40].

Within the first week, hMSCs in both propagation and osteogenic differentiation medium continue to proliferate, forming a culture consisting of a few cell layers. In comparison, those maintained in adipogenic differentiation medium were observed to continue to proliferate only to monolayer confluence regardless of oxygen tension. In adipogenic differentiation, growth arrest, which can be mediated by cell contact inhibition when cells reach confluence, is a prerequisite for the initiation of differentiation [41]. The progression from proliferation to resting state by cells in propagation medium is reflected by the gradual increase in the redox ratio observed from both HP an NP cells. hMSCs grown in differentiation medium continue towards a differentiated state after proliferation and a correlation between the maintenance of a low redox ratio and differentiation associated activity can be made.

The initial redox ratio increase of HA hMSCs was accompanied by minimal proliferation to complete confluence, and not multiple cell layers, indicating that the cells reached a state of low activity prior to their delayed differentiation response. At 20% oxygen, a strong response to adipogenic differentiation factors was reflected by the decreased redox ratio of NA cells and the observation of cellular morphology using TPEF images similar to those in Figure 4D and 4H, in which lipid droplet accumulation was clearly visible from day 8 onward. In Figure 8, transmission images from the final day of experimentation illustrate the different extent of lipid droplet accumulation between HA and NA cells. The greater redox ratio of the HA cells compared to the NA cells and the lack of large lipid droplet accumulation indicate that adipogenic differentiation is decreased at 5% oxygen. Inhibition of adipogenic differentiation by hypoxia has been reported previously[4] and may be due to decreased expression of PPARγ, a transcription factor necessary for adipogenesis, induced by hypoxia [42]. Indeed, LPL mRNA, a transcription target of PPARγ [43], is expressed at significantly lower levels in HA cells than it is in NA cells (Figure 9D). We can therefore correlate adipogenic differentiation with an increase in metabolic activity, which we measure via redox ratio imaging. An increase in metabolic activity in conjunction with adipogenic differentiation would be in agreement with studies that report an up regulation of the TCA cycle in murine 3T3-L1 cells during adipogenic differentiation[38] as well as increased oxygen consumption from mesenchymal cells differentiated into adipose tissue[44].

In this study, we monitor osteogenic differentiation from hMSCs in both normoxic and hypoxic conditions via the maintenance of a lower redox ratio, up-regulation of BSP mRNA, the expression and deposition of type 1 collagen and mineral staining by the alizarin red assay. We observe statistically lower redox ratios from hMSCs in osteogenic differentiation medium than we do from those in propagation medium. A similar increase in metabolic activity has been reported (via MTT assay) for embryonic stem cells in osteogenic differentiation medium at 20% oxygen by Karner et al. [45]. Many studies have shown that hypoxia can inhibit osteogenic differentiation [46]–[48]. However, recent experiments have demonstrated that hMSCs constantly maintained in hypoxic conditions, as they are in this study, retain the ability to differentiate into an osteoblast phenotype [49], [50]. In agreement with other studies[50], [51], we have found that osteogenic differentiation is more advanced under normoxia than hypoxia.[49], [50]. This observation is based on the recording of a lower redox ratio from NO cells (compared to HO) throughout the experiment as well as greater expression of BSP and COL1 mRNA, and higher levels of alizarin red staining on the final day of culture.

While by day 16 we observe greater COL1a1 mRNA expression from osteogenically differentiating cells under normoxic conditions in concordance with reports in the literature [45]–[47], COL1a1 mRNA expression is higher on day 8 for the hypoxic group, suggesting that collagen production may start earlier on for these cells. Indeed, collagen type 1 deposition appears to develop more rapidly in the hypoxia group as observed by SHG imaging (Figure 6). No collagen deposition is observed via SHG from cells in propagation medium (not shown) despite their expression of COL1a1 and COL2a1 mRNA. This lack of collagen deposition may be due to the absence of Vitamin C in the propagation medium, which is required for collagen production[51] or as Liu et al. speculate, due to changes in either the expression of protein or its intracellular vs extracellular localization [52].Whatever the mechanism, we are able to conclude from these observations that SHG is a much more direct method of characterizing the production and deposition of fibrous collagens than mRNA expression alone.

### Oxidative stress, lipofuscin and oxygen tension

Lipofuscin has been identified in the literature as a marker of aging in response to oxidative stress. The accumulation of lipofuscin has been shown by others to respond to oxygen tension, incubator temperature and addition of antioxidants to the culture medium [5], [53]. It is clear from our data that culturing hMSCs at 20% oxygen can lead to dramatic lipofuscin accumulation, while growth at 5% oxygen provides some protection. The hMSCs in each oxygen concentration group are derived from the same donor bone marrow isolated at 20% oxygen. It is difficult to know how much lipofuscin was present in the initial donated bone marrow. However, initial observations of hMSCs after two, one week expansions in hypoxic conditions indicate only a low level of lipofuscin present in each cell.

In a model proposed by Sitte et al. [54], average cellular lipofuscin levels can be described by a combination of cellular proliferative rate and lipofuscin accumulation rate. In proliferating populations, average lipofuscin levels are maintained by the dilution of accumulated lipofuscin to each daughter cell during division. As cultures reach confluence and the proliferation rate decreases, steady, or dramatic increases in average lipofuscin levels are observed with maintenance or acceleration in the rate of lipofuscin accumulation, respectively. Thus, isolation of hMSCs at 20% oxygen may induce, or maintain a somewhat balanced accumulation and dilution (through cell division) of lipofuscin, while expansion at 5% oxygen sees average lipofuscin dilution and minimal or no accumulation leading to the lower observed lipofuscin concentration at the end of two passages. In addition, one can conclude that maintaining hMSC cultures at 5% oxygen leads to a reduction in the rate of lipofuscin accumulation as we do not observe continued proliferation from hMSCs under hypoxic conditions.

While the mechanism for lipofuscin formation and accumulation is not yet clear, evidence supports a role for reactive oxygen species (ROS) originating from autophagocytosed mitochondria [55]. As mitochondria age and become defective they are degraded along with other cell components in the lysosomes, where mitochondrial ROS in the form of H_2_O_2_ can react with other lysosomally located components leading to lipofuscin formation. Lipofuscin accumulation in turn leads to dysfunctional lysosomes (as it is not degraded or exported from the cell) and a positive feedback loop in which decreased turnover of dysfunctional mitochondria results in more lipofuscin production [55].

The lower rate of lipofuscin accumulation observed at 5% oxygen may therefore be due to oxygen limited oxidative phosphorylation and an overall reduction in ROS production, which has been observed in cultures maintained at low oxygen levels [56]. Furthermore, it has been reported that osteogenic differentiation leads to an up-regulation of intrinsic cellular antioxidant enzymes [37] and a reduction in cellular ROS, which may account for our observation that hMSCs in osteogenic differentiation medium accumulate the least amount of lipofuscin. Conversely, the increased use of oxidative phosphorylation, and therefore ROS production [55], during adipogenic differentiation [38] may account for the increase in lipofuscin observed in NA cells. However, as we have previously stated, undifferentiated hMSCs preferentially make use of glycolysis, which should have the effect of reducing the amount of intracellular ROS produced, confounding our observation of equally dramatic lipofuscin accumulation from cells in propagation medium under atmospheric oxygen tension. If lipofuscin is indeed formed in response to cellular ROS, it is possible that isolation and maintenance of hMSC at 20% oxygen leads to premature aging, accumulation of defective mitochondria and subsequent lipofuscin formation, while the cells themselves are producing ATP via glycolysis.

This finding is important and supports observations of increased ROS, lipofuscin and decreased differentiation potential from aged mesenchymal stem cells [57]. In addition it is now clear in the literature that growth of hMSCs at low oxygen tensions maintains their differentiation capacity[50], [58], which may be due to a reduction in oxidative stress induced aging [5].

### Two-photon microscopy

This study demonstrates that it is possible to monitor the metabolic activity, morphology, ECM production and oxidative stress of hMSCs in a non-invasive manner. The MPM based monitoring methods in this study were developed with an “off the shelf” commercial system and simple data analysis techniques with the intent of providing a method that could be widely adopted by laboratories with a diversity of comparable equipment. This method therefore represents a powerful tool, which enables researchers to monitor engineered tissues and optimize culture conditions in a near real time manner. While we have demonstrated this technique in simple, two dimensional cultures, other studies have illustrated the power of MPM based techniques for imaging intact, three dimensional tissues [59]–[61] and we are confident that this technique can be readily adopted in a similar manner. Current MPM technologies are limited to relatively shallow imaging depths of up to approximately 1 mm [11] and by the geometry of the microscope, which can restrict the nature of the sample to be evaluated. However, as fiber optic based MPM systems develop [62], [63], it is reasonable to conclude that more flexible imaging geometries will be possible, enabling incorporation of MPM monitoring into bioreactors used in tissue engineering.

### Conclusion

This study describes the use of TPEF and SHG microscopy for the monitoring of changes in the intrinsic cellular fluorescence and non-linear scattering associated with differentiation and oxidative stress in cultures of human mesenchymal stem cells. We find that the redox ratio based on the TPEF of NAD(P)H and FP can be used repeatedly as a non invasive estimate of differentiation related metabolic activity. Interestingly, distinct patterns of change in the redox ratio are observed for stem cells undergoing adipogenic and osteogenic differentiation. The fluorescence of other intrinsic fluorophores, such as lipofuscin, can be tracked over time as markers of cellular biochemical status and oxidative stress. In addition, TPEF and SHG images of hMSCs undergoing differentiation can be used to monitor cell morphology (TPEF) and the deposition of fibrous collagens (SHG) without the need for invasive processing or significant disruption of the culture conditions. Therefore, we can conclude that this implementation of multiphoton microscopy is indeed a powerful, informative tool for monitoring the development of engineered tissues.

## Materials and Methods

### Chemicals and Media components

Chemicals, unless otherwise noted, were purchased from Sigma-Aldrich (St. Louis, MO).

Propagation culture media components were purchased from Invitrogen (Carlsbad, CA).

### Cell culture

Human mesenchymal stem cells were obtained from Cambrex (East Rutherford, NJ) and isolated, as previously described [64], on tissue culture plastic at atmospheric oxygen (∼20%) and stored under liquid nitrogen as Passage 0 (P0). Subsequently, hMSCs were thawed and passaged thrice under 5% oxygen and plated for experimentation. For our studies, hMSCs were plated at a density of 150,000 cells per 35 mm dish. The base culture medium, also referred to as propagation medium, consisted of Dulbecco's Modified Eagle Medium (DMEM) without phenol red supplemented with 10% fetal bovine serum, 0.1 mM nonessential amino acids, 100 U/mL penicillin and streptomycin with a final glucose concentration of 4 g/L. To induce adipogenic differentiation, the following supplements were added to the medium: 0.5 mM 3-isobutyl-1-methyl-xanthine, 1 µM dexamethasone, 5 mg/ml insulin, and 50 mM indomethacin. For osteogenic differentiation, the propagation medium was supplemented with 100 nM dexamethasone, 10 mM b-glycerophosphate, and 0.05 mM l-ascorbic acid-2-phosphate. All experimental groups were maintained in a humidified incubator at 37 C and 5% CO_2_ at either uncontrolled (atmospheric ∼20%) oxygen tension, or at 5% controlled oxygen tension. Culture medium was changed every 3 days for either 16 or 21 days. Due to the observation that cells undergoing osteogenic differentiation often peel off the culture dish[65], [66], hMSCs in osteogenic differentiation medium were only cultured for 16 days to ensure accurate data. Care was taken such that the 5% oxygen experimental group spent no more than 4% (∼20 hrs/22 days) of the total experiment in atmospheric oxygen including time for imaging and medium exchanges.

### KCN- FCCP treatments

To inhibit oxidative phosphorylation and increase the mitochondrial concentration of NADH (and decrease oxidized FP), potassium cyanide (KCN) in PBS was added to the culture medium for a final concentration of 4 mM. Addition of 4 µM (final) carbonyl cyanide 4 (trifluoromethoxy) phenylhydrazone (FCCP) was used to produce an inverse effect, decreasing the mitochondrial NADH fluorescence and increasing FP oxidation (and thus fluorescence). TPEF spectra and images at excitations of 755 nm and 860 nm were acquired before and after treatment with either KCN or FCCP.

### RT-PCR

RNA was collected from experimental replicates on each day of imaging with the RNAeasy mini kit (Qiagen, Valencia, CA). DNA was obtained via reverse transcription using the High Capacity cDNA archive kit (Applied Biosystems, Foster City, CA) and the expression of the differentiation markers were examined relative to GapDH using commercially available primers from Applied Biosystems: lipoprotein lipase (LPL Product #Hs01012571_m1), bone sialoprotein(BSP Product #Hs00173720_m1), type 1 collagen (COL1a1 Product #Hs01076777_m1) type 2 collagen (COL2a1 Product #Hs01060345_m1) and GapDH (Product #Hs99999905_m1).

### Colorimetric Stains for Mineralization and Lipid Accumulation

Mineral precipitation was determined by Alizarin red staining as previously described [67]. Briefly, dishes on day 16 were fixed in 10% formalin for 20 minutes, washed and stained with a 40 mM Alizarin red (pH 4.2) solution. After incubating for 30 minutes at room temperature, dishes were washed and observed under phase microscopy. To quantitatively assess the level of Alizarin red staining, dishes were incubated with 10% Acetic acid for 30 minutes after which the contents of the dish were collected into 2 mL sample tubes, vortexed, heated to 85 C for 10 minutes, cooled on ice for 5 minutes and pH neutralized with 10% ammonium hydroxide. After centrifugation (20,000 g, 15 min), the absorption of the supernatant at 405 nm was assessed in triplicate in a clear 96 well plate with a Versamax microplate reader (Molecular Devices).

To assess lipid accumulation, hMSCs were stained with an Oil Red - O solution as previously described [2]. Briefly, dishes were fixed for 20 minutes in 10% formalin, washed and incubated with a fresh 60% Oil Red -O solution in DI water for 45 minutes followed by washing to remove excess stain. Stained dishes were then observed in transmission mode on a Leica DMIRE2 confocal microscope using the 543 nm laser. Oil Red - O stain was eluted from the stained dishes with pure ethanol and the absorbance at 490 nm was determined in triplicate in a clear 96 well plate with a Versamax microplate reader.

### Confocal and Multiphoton microscopy

Confocal or two photon excited fluorescence (TPEF) and second harmonic generation (SHG) micrographs were acquired on a Leica (Wetzlar, Germany) DMIRE2 microscope with a TCS SP2 scanner. The system was equipped with a 63x (NA 1.2) water immersion objective, which produced images with a 238 by 238 µm field of view. The external excitation light source was a Mai Tai tunable (710–920 nm) titanium sapphire laser emitting 100 fs pulses at 80 MHz (Spectra Physics, Mountain View CA). Samples were maintained on 35 mm culture dishes with number 1.5 cover glass bottoms (MATTEK, Ashland MA) and placed in a micro incubator (DH-35i) (Warner instruments, Hamden CT) on the microscope stage to ensure that the temperature remained at 37 C. Images and/or spectra were excited with 755 nm and, 860 nm (TPEF) or 800 nm (SHG). Fluorescence emission spectra were detected using the TCS SP2 scanner and the Leica LCS software from 400 nm to 700 nm in 20 steps with a 15 nm detector bandwidth. TPEF images were acquired simultaneously by two non-descanned PMTs with a filter cube containing a 700 nm short pass filter (Chroma SPC700bp) a dichroic mirror (495dc), and two bandpass filters centered at 455 nm (Chroma 455bp70) and 525 nm (Chroma 525bp50). SHG was collected through a filter centered at 400 nm (Chroma hq400/20m-2p). The average power measured at the sample for two photon excitation was approximately 20 mW for both 755 nm and 860 nm and 50 mW for 800 nm (SHG). Confocal images of hMSCs stained with Lysotracker Red (excitation 554 nm, emission 600–650 nm) or Mitotracker Orange (excitation 554 nm, emission 575–625 nm) (Invitrogen) were obtained by following the manufacturer's instructions. Analysis was performed with the Leica Confocal Software (Leica) and Matlab (Mathworks Natick MA).

### Correction of pixel value to fluorescence intensity

Images acquired on the Leica LCS system at 8 bits per pixel were converted in Matlab from pixel value to a fluorescence intensity value so that, at a given power, changes in fluorescence intensity could be correlated linearly to changes in fluorophore concentration for a number of PMT offset and gain settings in order to compare data acquired from multiple days and experiments. This pixel fluorescence intensity to fluorophore concentration transfer function was determined for each PMT gain and offset setting used in the study using a standard curve of fluorescein solution of known concentration. The fluorescence intensity images were then normalized by the square of the excitation laser power, at the sample, to minimize day to day fluctuations in laser power. The resulting image was then presented in relative fluorescein concentration units.

### Spectral analysis

TPEF emission spectra were acquired with the Leica LCS software as a series of 20 images spanning 400 to 700 nm in 15 nm increments. The cellular TPEF emission spectra for each microscopic field was extracted from these images, after conversion to concentration units and manual selection of a region of interest (ROI) containing the cells and rejecting most of the (non-cell) background. The resulting 20 point arbitrary concentration units vs wavelength (nm) spectrum was then interpolated to 301 points (400–700 nm) and smoothed with a 40 point moving average. Linear unmixing of the spectra was accomplished using the ALS script from the PLS 3.5 Matlab toolbox (Eigenvector Research, Wenatchee WA). This script uses an alternating least squares algorithm to optimize the fit to spectral data given at a minimum the number of spectral components and initial guesses about either the relative weight or spectral shape of the components [68], [69]. To identify the spectral components of fluorescence, interpolated and smoothed TPEF emission spectra were normalized to a maximum value of 1. First, TPEF emission spectra (ex 755 nm and 860 nm) from cells with no apparent bright punctuate fluorescence were unmixed with non-negativity constraints and no initial guess of spectral component shapes. The two resulting components were then used to analyze the TPEF emission spectra from cells containing bright punctuate auto fluorescence in order to find any additional spectral components of fluorescence. This was accomplished by supplying the two found components as strict values, and additional (up to 3) components as unknowns, all with non-negativity constraints and equal initial weights. In this manner it was found that the spectral analysis was most meaningful when attempted with three basis components, the two acquired from the initial cell analysis and a third component that was prominent in cells with bright punctuate fluorescence.

### Image analysis

The collagen content in SHG images was estimated as previously described[70]. Briefly, SHG images were converted to binary format after using an adaptive threshold and collagen density was displayed as a percent value of “on” pixels in the SHG image. Prior to analysis, TPEF images were converted from pixel values to arbitrary concentration units as described above. Random noise was suppressed via a 2 by 2 median filter. A three color (RGB) concentration image was then calculated from the data obtained at 755 and 860 nm TPEF excitation such that the pixel by pixel calculated concentration of each fluorophore, NAD(P)H, FP and Lipofuscin was stored in the red, green, and blue channels respectively. To isolate the contributions from each of the fluorophores in the TPEF images, and calculate the concentration images it was assumed that NAD(P)H was not excited at 860 nm, lipofuscin contributed to both 455 nm and 525 nm channels at 755 and 860 nm excitations and that FP fluorescence contributed to the 455 nm channel regardless of excitation wavelength. Imaging an aqueous solution of FAD (Sigma) confirmed that there was minimal FP fluorescence crosstalk (2%) between the 455 nm and 525 nm channels. The concentrations of each fluorophore were determined based on the procedure described below.

Lipofuscin fluorescence was the major contributor to the 455 nm channel at 860 nm excitation.
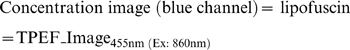



A binary morphological mask (MASK) was then created using the data in the 455 nm channel at 860 nm excitation such that the MASK represented the location of the lipofuscin fluorescence determined as all pixels with a value above a threshold of 5 (2% of 255 for 8 bit images) to eliminate the maximum possible FP contribution to the 455 nm channel. Multiplication of the remaining images by the MASK (lipofuscin fluorescence was represented by a 0, and all other pixels by a 1), served to morphologically eliminate the lipofuscin fluorescence in these images. This was possible because lipofuscin fluorescence is limited to the lysosomes (as confirmed by colocalization studies described below). The FP concentration image could therefore be determined from the 525 nm channel at 860 nm excitation.
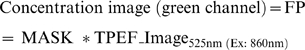



Similarly, the NAD(P)H concentration was determined from the 455 nm channel at 755 nm where the FP contribution is negligible.
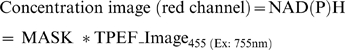



The mean concentration of each fluorophore per cell was determined by hand-defining an ROI outlining each cell and calculating the mean concentration for each channel (RGB:NAD(P)H, FP, Lipofuscin). As an estimate of cellular metabolic activity, the redox ratio was calculated as:
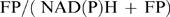



The redox ratio was determined on a cell by cell basis from the data obtained as described above.

### Statistics

Differences in reported mean values were tested for significance using a two tailed students t-test to a p value of 0.05 or less, unless otherwise noted.
